# Front-line Experiences and Perspectives of Older and Younger Registered Nurses during the COVID-19 Pandemic

**DOI:** 10.1093/geroni/igaa057.3467

**Published:** 2020-12-16

**Authors:** Victoria Raveis, Nancy VanDevanter, Christine Kovner, Gary Yu, Laura Jean Ridge, Kimberly Glassman

**Affiliations:** 1 New York University, Maplewood, New Jersey, United States; 2 NYU Rory Meyers College of Nursing, New York, New York, United States; 3 Rory Meyers College of Nursing, New York, New York, United States; 4 University of Michigan, Ann Arbor, Michigan, United States; 5 New York University, NY, New York, United States

## Abstract

Having an experienced and trained healthcare workforce available during public health emergencies, such as the COVID-19 pandemic, is critical. While all healthcare workers are at risk of contracting COVID-19, older workers are at increased risk of serious or fatal illness. This investigation explores the front-line experiences and perspectives of registered nurses (RNs) at a major New York City medical center during the COVID-19 pandemic, focusing on two age cohorts: older (50+) and younger (20-49) RNs. An anonymous internet-based survey was sent to all RN’s employed at the center. Data collection was initiated in May 2020, following the initial surge in NYC’s COVID-19 related hospitalizations and deaths; 1,483 surveys were completed. This investigation found that in comparison to younger RNs (n=1,067), older RNs’ (n=416) psychosocial well-being was significantly better -- fewer depression, anxiety, and PTSD symptoms (p<.001). They were less stressed caring for COVID-19 patients (p <.001) and less worried about work-related exposure risk (p<.001). They also reported higher job satisfaction (p<.001), less work-home stress (p<.001), a higher commitment to choosing the nursing profession (p<.001), were more confident in the profession (p<.001) and to meeting its’ expectations (p<.001). Overall, although older RNs represent a smaller proportion of the nursing workforce, their collective expertise and clinical experience in healthcare delivery are significant. The older RN workforce is a seasoned resource to draw upon during public health emergencies and a valuable role model for younger RNs, particularly given their continued adherence to nursing, during this COVID-19 time of uncertainty and personal health risk.

